# Risk factors for severe radiation-induced dermatitis in nasopharyngeal carcinoma patients undergoing radiotherapy

**DOI:** 10.3389/fonc.2026.1851374

**Published:** 2026-07-27

**Authors:** Dong-Xia Liao, Dan Zhao, Feng-Ling Yang, Zhang Li, Xiao-Xue Deng, Zi-Dan Qiu

**Affiliations:** 1 Department of Radiation Oncology, Longyan First Affiliated Hospital of Fujian Medical University, Longyan, Fujian, China; 2 Department of Oncology, Longyan First Affiliated Hospital of Fujian Medical University, Longyan, Fujian, China

**Keywords:** albumin, C-reactive protein, nasopharyngeal carcinoma, neutrophil-to-lymphocyte ratio, radiation-induced dermatitis, radiotherapy, risk factors

## Abstract

**Background:**

Radiation-induced dermatitis (RID) is a common toxicity in patients with nasopharyngeal carcinoma (NPC) undergoing radiotherapy. This study aimed to evaluate the incidence, onset time, and factors associated with severe RID in patients with NPC.

**Methods:**

This retrospective study included 287 patients with histologically confirmed NPC who received radiotherapy between August 2020 and August 2025. RID was graded according to the Radiation Therapy Oncology Group (RTOG) acute radiation morbidity criteria. Severe RID was defined as RTOG grade ≥3. Patients were classified into non-severe RID (RTOG grade 0–2) and severe RID groups. Baseline, clinicopathological, treatment-related, and laboratory variables were compared between groups. Logistic regression was used to identify factors associated with severe RID.

**Results:**

Patients were stratified into a non-severe group (RTOG grade 0–2, n = 201) and a severe group (RTOG grade ≥3, n = 86). The mean onset time was 24.8 ± 6.7 days for RTOG grade ≥1 RID and 37.6 ± 6.3 days for RTOG grade ≥3 RID. In multivariable analysis, older age (odds ratio [OR] = 1.03, 95% confidence interval [CI]: 1.01–1.06), smoking history (OR = 1.93, 95% CI: 1.11–3.55), higher total radiation dose (OR = 1.08, 95% CI: 1.01–1.15), elevated C-reactive protein (OR = 1.12, 95% CI: 1.06–1.20), higher neutrophil-to-lymphocyte ratio (OR = 1.53, 95% CI: 1.21–1.96), and larger irradiation area (OR = 1.16, 95% CI: 1.06–1.28) were positively associated with severe RID, whereas higher albumin was inversely associated with severe RID (OR = 0.89, 95% CI: 0.82–0.95).

**Conclusions:**

Severe radiation-induced dermatitis was observed in 30.0% of patients with nasopharyngeal carcinoma receiving radiotherapy. Older age, smoking history, higher radiation dose, elevated inflammatory status, larger irradiation area, and lower albumin level were independently associated with severe radiation-induced dermatitis.

## 1 Introduction

Nasopharyngeal carcinoma (NPC) is a distinct epithelial malignancy for which radiotherapy remains the cornerstone of curative treatment, owing to the anatomical location of the primary tumor and the radiosensitivity of the disease (1). With the widespread adoption of intensity-modulated radiotherapy, locoregional control has improved substantially; however, radiotherapy-related toxicities continue to affect treatment adherence, symptom burden, and quality of life (2, 3). Among these toxicities, radiation-induced dermatitis is one of the most common acute adverse events during head and neck irradiation. In clinical practice, skin reactions may range from faint erythema and dry desquamation to confluent moist desquamation and ulceration (4). More severe forms may cause pain, exudation, treatment interruption, and increased supportive care requirements (5, 6).

The development of radiation-induced dermatitis is multifactorial. Current evidence suggests that radiation-related skin injury reflects interactions among direct epithelial damage, impaired barrier function, local microvascular changes, and amplification of inflammatory signaling (7). Recent studies in head and neck cancer have shown that skin barrier characteristics, thermal changes during treatment, and baseline hematologic or inflammatory indicators may be associated with subsequent dermatitis severity (8). In parallel, emerging predictive studies have increasingly incorporated dosimetric, radiomic, and machine-learning approaches, underscoring the need to move beyond descriptive toxicity reporting toward individualized risk stratification (9–11). This issue is particularly relevant in NPC. Patients with NPC frequently receive high-dose radiotherapy to complex target volumes involving the nasopharynx and bilateral cervical lymphatic drainage regions, often in combination with concurrent chemotherapy. Consequently, a relatively large area of cervical skin may be exposed to clinically meaningful radiation doses (12, 13). Although radiation-induced dermatitis is a well-recognized toxicity during radiotherapy for nasopharyngeal carcinoma, the factors associated with clinically relevant severe dermatitis remain incompletely characterized. Previous studies have reported patient-related or treatment-related factors, such as smoking, nutritional status, body mass index, comorbidities, radiation technique, and dose-related parameters, but these factors have often been evaluated separately or within specific treatment settings (14, 15). In particular, limited evidence is available on the joint evaluation of routinely obtainable clinical characteristics, inflammatory and nutritional markers, radiotherapy planning parameters, and selected systemic treatment variables in characterizing the risk profile of severe radiation-induced dermatitis in patients with nasopharyngeal carcinoma (16, 17).

Therefore, this study aimed to investigate clinically accessible factors associated with severe radiation-induced dermatitis in patients with nasopharyngeal carcinoma undergoing radiotherapy, thereby providing evidence to support risk stratification in routine clinical practice.

## 2 Methods

### 2.1 Study design

This retrospective study enrolled patients with histologically confirmed nasopharyngeal carcinoma who underwent radiotherapy at our institution between August 2020 and August 2025. Eligible patients were aged 18 years or older, had complete clinicopathological and treatment data, and received standardized radiotherapy with or without concurrent chemotherapy at our center. Patients were included if radiation-induced dermatitis could be reliably assessed and graded throughout the course of treatment based on documented medical records. The exclusion criteria were as follows: prior radiotherapy to the head and neck region, history of other synchronous malignant tumors, pre-existing severe skin disorders or active skin infection in the irradiation field, interruption or discontinuation of radiotherapy for non-medical reasons, and incomplete clinical, dosimetric, or follow-up data. Informed consent was obtained from all participants. The study protocol was reviewed and approved by the Ethics Committee of our hospital. All procedures were conducted in accordance with relevant guidelines and regulations and complied with the ethical principles of the Declaration of Helsinki. All data were anonymized prior to analysis to ensure confidentiality and protect participant privacy.

### 2.2 Outcome definition and grouping

The primary study outcomes were the occurrence and severity of radiation-induced dermatitis during the course of radiotherapy. Radiation-induced dermatitis was assessed and graded according to the Radiation Therapy Oncology Group (RTOG) acute radiation morbidity scoring criteria. Specifically, grade 0 indicated no visible skin change; grade 1 was defined as faint or dull erythema, dry desquamation, epilation, or decreased sweating; grade 2 as bright erythema, patchy moist desquamation, or moderate edema; grade 3 as confluent moist desquamation outside skin folds or pitting edema; and grade 4 as ulceration, hemorrhage, or necrosis. Skin reactions were evaluated at regular intervals throughout radiotherapy and at the completion of treatment on the basis of clinical examination and medical record documentation. All dermatitis assessments were performed independently by trained clinicians or radiation oncologists according to standardized grading procedures.

Radiation-induced dermatitis occurrence was defined as the development of any documented skin reaction of RTOG grade ≥1 during radiotherapy. The overall incidence of radiation-induced dermatitis was calculated as the proportion of patients with RTOG grade ≥1 dermatitis among the total study population. Severe radiation-induced dermatitis was defined as RTOG grade ≥3 dermatitis. The onset time of radiation-induced dermatitis was defined as the interval, in days, from the initiation of radiotherapy to the first documented occurrence of RTOG grade ≥1 dermatitis. For patients who developed severe dermatitis, the onset time of severe radiation-induced dermatitis was defined as the interval from the initiation of radiotherapy to the first documented occurrence of RTOG grade ≥3 dermatitis.

To facilitate risk factor analysis for clinically relevant severe skin toxicity, patients were stratified into a non-severe dermatitis group (RTOG grade 0–2) and a severe dermatitis group (RTOG grade ≥3). This grouping approach was adopted because RTOG grade ≥3 dermatitis represents confluent moist desquamation, pitting edema, or more serious skin injury that generally requires closer monitoring and more intensive clinical management.

### 2.3 Data collection

Clinical data were retrospectively extracted from the electronic medical record system, radiotherapy planning system, medication administration records, and laboratory database of our institution using a standardized data collection form. The collected variables included demographic characteristics, clinicopathological features, treatment-related parameters, laboratory findings, and dermatitis-related outcome data.

Demographic data included age, sex, body mass index (BMI), smoking history, and alcohol consumption history. Clinicopathological variables included T stage, N stage, overall TNM stage, histopathological type, and comorbid conditions. Indicators reflecting nutritional status, including serum albumin and hemoglobin levels, were also collected where available.

Treatment-related variables included radiotherapy modality, total prescribed radiation dose, dose per fraction, duration of radiotherapy, irradiation area, and whether concurrent chemotherapy was administered. For patients who received concurrent chemotherapy, the chemotherapy regimen and number of treatment cycles were recorded. In addition, the use of concurrent targeted therapy was collected, including the type of targeted agent, such as cetuximab or nimotuzumab. Immunotherapy-related variables were also extracted, including any immunotherapy and the treatment phase in which immunotherapy was administered, categorized as induction immunotherapy, concurrent immunotherapy, or adjuvant/consolidation immunotherapy. These immunotherapy categories were not mutually exclusive.

Dermatitis-related data included the maximum RTOG grade recorded during radiotherapy, the occurrence of RTOG grade ≥1 dermatitis, the occurrence of RTOG grade ≥3 dermatitis, the date of first recorded RTOG grade ≥1 dermatitis, and the date of first recorded RTOG grade ≥3 dermatitis when applicable. Laboratory variables were obtained from baseline examinations performed before initiation of radiotherapy and included routine hematological parameters, inflammatory markers, and nutritional indicators. Hematological parameters included white blood cell count, neutrophil count, lymphocyte count, platelet count, and hemoglobin level. Inflammatory and nutritional markers were collected according to data availability in the medical records and were included in the subsequent analysis as potential predictors of severe radiation-induced dermatitis.

### 2.4 Data quality control

To ensure data accuracy and reliability, all study variables were extracted independently by two investigators using a standardized data collection form. After the initial extraction, the two datasets were cross-checked to verify the consistency and accuracy of the recorded information. Any discrepancies identified during the review process were resolved through discussion, and when consensus could not be reached, adjudication was performed by a third senior investigator. The case selection process was based on explicit inclusion and exclusion criteria to ensure a clear and reproducible patient screening pathway. The outcome assessment was conducted using unified RTOG grading criteria for radiation-induced dermatitis to reduce interobserver variability and outcome misclassification. For dermatitis-related outcomes, the maximum RTOG grade and the first documented dates of RTOG grade ≥1 and RTOG grade ≥3 dermatitis were verified against longitudinal radiotherapy records, clinical notes, and nursing documentation when available. For treatment-related variables, chemotherapy, targeted therapy, and immunotherapy records were cross-checked using medication administration records and treatment summaries. Missing data were handled according to predefined principles. Variables with a low proportion of missing values were managed by case-wise exclusion from the corresponding analyses. For variables with a relatively high proportion of missing data, multiple imputation was considered when appropriate and statistically justifiable; otherwise, such variables were excluded from the final analysis to avoid introducing substantial bias. The extent and handling of missing data were documented during the analytical process.

### 2.5 Statistical analysis

All statistical analyses were performed using IBM SPSS Statistics, version 30.0 (IBM Corp., Armonk, NY, USA). Continuous variables were summarized as mean ± standard deviation or median with interquartile range, as appropriate, and categorical variables were presented as frequencies and percentages. Between-group comparisons were performed using the independent-samples t test for continuous variables and the chi-square test for categorical variables. The incidence, severity distribution, and onset time of radiation-induced dermatitis were described in the overall cohort. Univariate logistic regression analysis was used to evaluate the associations between candidate variables and severe radiation-induced dermatitis, defined as RTOG grade ≥3. Variables showing statistical significance in the univariate analysis were considered for multivariable logistic regression modeling. The final multivariable model was constructed according to statistical significance, clinical relevance, potential collinearity among candidate variables, and model stability. Results were reported as odds ratios (ORs) with 95% confidence intervals (CIs). Subgroup analyses were conducted according to age group, concurrent chemotherapy status, TNM stage, and nutritional status. Interaction P values were estimated by adding corresponding interaction terms to the regression models. Additional sensitivity models were constructed by extending the main multivariable model to include concurrent chemotherapy and advanced TNM stage separately and simultaneously. Multiple imputation was also performed to assess the robustness of the complete-case results. All tests were two-sided, and P < 0.05 was considered statistically significant.

## 3 Results

### 3.1 Patient selection and grouping

A total of 313 patients with nasopharyngeal carcinoma who received radiotherapy at our institution between August 2020 and August 2025 were initially screened for eligibility. After application of the predefined inclusion and exclusion criteria, 26 patients were excluded for the following reasons: age younger than 18 years (n = 3), previous head and neck radiotherapy (n = 5), concomitant synchronous malignant tumors (n = 4), interruption or failure to complete radiotherapy for non-medical reasons (n = 6), and incomplete key clinical, dosimetric, or follow-up data (n = 8). Ultimately, 287 patients were included in the final analysis. For outcome assessment, all included patients were evaluated for the occurrence and severity of radiation-induced dermatitis during radiotherapy according to the RTOG acute radiation morbidity scoring criteria. Radiation-induced dermatitis was graded according to the RTOG acute radiation morbidity scoring criteria. Among the 287 included patients, 28 (9.8%) had RTOG grade 0 dermatitis, 76 (26.5%) had grade 1, 97 (33.8%) had grade 2, 82 (28.6%) had grade 3, and 4 (1.4%) had grade 4. Overall, 259 patients developed RTOG grade ≥1 dermatitis, corresponding to an overall incidence of 90.2%. Severe radiation-induced dermatitis, defined as RTOG grade ≥3, occurred in 86 patients, corresponding to an incidence of 30.0%. Among patients with RTOG grade ≥1 dermatitis, the mean time to first recorded dermatitis was 24.8 ± 6.7 days after initiation of radiotherapy. Among patients with severe dermatitis, the mean time to first recorded RTOG grade ≥3 dermatitis was 37.6 ± 6.3 days (**Supplementary Table 1**). For the severity-based analysis, patients were stratified into a non-severe radiation-induced dermatitis group (RTOG grade 0–2, n = 201) and a severe radiation-induced dermatitis group (RTOG grade ≥3, n = 86). This grouping was used for subsequent comparative and risk factor analyses.

### 3.2 Baseline demographic and clinicopathological characteristics

A total of 287 patients were included in the baseline comparison, including 201 patients in the non-severe radiation-induced dermatitis group and 86 patients in the severe group. Patients in the severe group were significantly older than those in the non-severe group (51.9 ± 11.4 vs. 47.3 ± 10.8 years, t = -3.25, P = 0.001). The severe group also had a lower BMI (21.7 ± 3.4 vs. 22.9 ± 3.1 kg/m², t = 2.92, P = 0.004) and a higher proportion of patients with a smoking history (38.4% vs. 23.9%, χ² = 6.24, P = 0.012). No significant between-group differences were observed in sex distribution or alcohol consumption history (both P > 0.05). With respect to clinicopathological characteristics, the severe group showed a more advanced tumor burden. Significant differences were observed between the two groups in T stage (χ² = 10.54, P = 0.015), N stage (χ² = 13.23, P = 0.004), and TNM stage (χ² = 11.81, P = 0.008). In particular, the proportions of T4, N2–N3, and stage III–IVA disease were higher in the severe group than in the non-severe group. By contrast, pathological type was similarly distributed between groups (χ² = 0.60, P = 0.740). In addition, comorbidities were more common among patients with severe radiation-induced dermatitis than among those with non-severe dermatitis (43.0% vs. 27.9%, χ² = 6.32, P = 0.012) (**Table 1**).

**Table 1:** Comparison of baseline demographic and clinicopathological characteristics between the non-severe and severe radiation-induced dermatitis groups.

Variable	Non-severe group (RTOG 0–2, n = 201)	Severe group (RTOG ≥3, n = 86)	Test statistic	P value
Demographic characteristics				
Age, years	47.3 ± 10.8	51.9 ± 11.4	t = -3.25	0.001
Male sex, n (%)	123 (61.2)	57 (66.3)	χ² = 0.67	0.414
BMI, kg/m²	22.9 ± 3.1	21.7 ± 3.4	t = 2.92	0.004
Smoking history, n (%)	48 (23.9)	33 (38.4)	χ² = 6.24	0.012
Alcohol consumption history, n (%)	42 (20.9)	27 (31.4)	χ² = 3.64	0.057
Clinicopathological characteristics				
T stage, n (%)			χ² = 10.54	0.015
T1	40 (19.9)	7 (8.1)		
T2	56 (27.9)	18 (20.9)		
T3	60 (29.9)	31 (36.0)		
T4	45 (22.4)	30 (34.9)		
N stage, n (%)			χ² = 13.23	0.004
N0	50 (24.9)	12 (14.0)		
N1	76 (37.8)	23 (26.7)		
N2	52 (25.9)	31 (36.0)		
N3	23 (11.4)	20 (23.3)		
TNM stage, n (%)			χ² = 11.81	0.008
I	20 (10.0)	4 (4.7)		
II	76 (37.8)	19 (22.1)		
III	84 (41.8)	47 (54.7)		
IVA	21 (10.4)	16 (18.6)		
Pathological type, n (%)			χ² = 0.60	0.740
Keratinizing squamous cell carcinoma	16 (8.0)	6 (7.0)		
Differentiated non-keratinizing carcinoma	42 (20.9)	15 (17.4)		
Undifferentiated non-keratinizing carcinoma	143 (71.1)	65 (75.6)		
Comorbidities, n (%)	56 (27.9)	37 (43.0)	χ² = 6.32	0.012

RTOG, Radiation Therapy Oncology Group; BMI, body mass index; TNM, tumor-node-metastasis.

### 3.3 Comparison of treatment-related characteristics and laboratory parameters

As shown in **Table 2**, several treatment-related characteristics and laboratory parameters differed between the non-severe and severe radiation-induced dermatitis groups. The distribution of radiotherapy modality differed significantly between the two groups (χ² = 8.30, P = 0.004). The severe group had a higher proportion of volumetric modulated arc therapy than the non-severe group. Compared with the non-severe group, the severe group had a higher total radiation dose (70.8 ± 4.1 vs. 69.4 ± 3.8 Gy, t = -2.79, P = 0.006), longer radiotherapy duration (47.9 ± 5.1 vs. 45.8 ± 4.6 days, t = -3.43, P < 0.001), and larger irradiation area (178.9 ± 27.3 vs. 166.5 ± 24.8 cm², t = -3.76, P < 0.001). Concurrent chemotherapy was more frequent in the severe group than in the non-severe group (86.0% vs. 71.1%, χ² = 7.25, P = 0.007). Among patients receiving concurrent chemotherapy, the number of chemotherapy cycles was also higher in the severe group (3.1 ± 1.1 vs. 2.7 ± 1.0, t = -2.70, P = 0.008). No significant between-group differences were observed in dose per fraction or chemotherapy regimen distribution. Concurrent targeted therapy was administered to 24 patients, including 16 patients in the non-severe group and 8 patients in the severe group. The proportion of concurrent targeted therapy did not differ significantly between groups (8.0% vs. 9.3%, χ² = 0.14, P = 0.707). The distribution of targeted therapy type, including cetuximab and nimotuzumab, was also comparable between groups (P = 0.932). Immunotherapy was administered to 33 patients, including 22 patients in the non-severe group and 11 patients in the severe group. No significant differences were observed between groups in any immunotherapy (10.9% vs. 12.8%, χ² = 0.20, P = 0.653), induction immunotherapy, concurrent immunotherapy, or adjuvant/consolidation immunotherapy. For laboratory parameters, the severe group had higher white blood cell counts, neutrophil counts, C-reactive protein levels, neutrophil-to-lymphocyte ratio, and erythrocyte sedimentation rate than the non-severe group, whereas lymphocyte counts, hemoglobin levels, albumin levels, and prealbumin levels were lower in the severe group (all P < 0.05). Platelet count did not differ significantly between the two groups (P = 0.108).

**Table 2:** Comparison of treatment-related characteristics and laboratory parameters between the non-severe and severe radiation-induced dermatitis groups.

Variable	Non-severe group (RTOG 0–2, n = 201)	Severe group (RTOG ≥3, n = 86)	Test statistic	P value
Treatment-related characteristics				
Radiotherapy modality, n (%)			χ² = 8.30	0.004
Intensity-modulated radiotherapy	155 (77.1)	52 (60.5)		
Volumetric modulated arc therapy	46 (22.9)	34 (39.5)		
Total radiation dose, Gy	69.4 ± 3.8	70.8 ± 4.1	t = -2.79	0.006
Dose per fraction, Gy	2.10 ± 0.12	2.13 ± 0.14	t = -1.84	0.066
Radiotherapy duration, days	45.8 ± 4.6	47.9 ± 5.1	t = -3.43	<0.001
Irradiation area, cm²	166.5 ± 24.8	178.9 ± 27.3	t = -3.76	<0.001
Concurrent chemotherapy, n (%)	143 (71.1)	74 (86.0)	χ² = 7.25	0.007
Chemotherapy regimen among patients receiving concurrent chemotherapy, n (%)			χ² = 1.18	0.553
Cisplatin alone	62 (43.4)	36 (48.6)		
Nedaplatin alone	56 (39.2)	29 (39.2)		
Cisplatin + fluorouracil-based regimen	25 (17.5)	9 (12.2)		
Number of chemotherapy cycles	2.7 ± 1.0	3.1 ± 1.1	t = -2.70	0.008
Concurrent targeted therapy, n (%)	16 (8.0)	8 (9.3)	χ² = 0.14	0.707
Type of concurrent targeted therapy, n (%)			χ² = 0.14	0.932
No targeted therapy	185 (92.0)	78 (90.7)		
Cetuximab	10 (5.0)	5 (5.8)		
Nimotuzumab	6 (3.0)	3 (3.5)		
Any immunotherapy, n (%)	22 (10.9)	11 (12.8)	χ² = 0.20	0.653
Induction immunotherapy, n (%)	12 (6.0)	6 (7.0)	χ² = 0.10	0.747
Concurrent immunotherapy, n (%)	7 (3.5)	4 (4.7)	χ² = 0.22	0.637
Adjuvant/consolidation immunotherapy, n (%)	8 (4.0)	4 (4.7)	χ² = 0.07	0.795
Laboratory parameters				
White blood cell count, ×10^9^/L	5.9 ± 1.4	6.4 ± 1.6	t = -2.65	0.008
Neutrophil count, ×10^9^/L	3.6 ± 1.1	4.2 ± 1.3	t = -4.00	<0.001
Lymphocyte count, ×10^9^/L	1.72 ± 0.46	1.43 ± 0.41	t = 5.05	<0.001
Platelet count, ×10^9^/L	238 ± 61	251 ± 66	t = -1.61	0.108
Hemoglobin, g/L	132.4 ± 14.8	126.1 ± 16.2	t = 3.21	0.002
C-reactive protein, mg/L	6.8 ± 3.9	10.7 ± 5.8	t = -6.65	<0.001
Neutrophil-to-lymphocyte ratio	2.24 ± 0.91	3.18 ± 1.27	t = -7.08	<0.001
Erythrocyte sedimentation rate, mm/h	22.6 ± 11.5	28.9 ± 13.4	t = -4.04	<0.001
Albumin, g/L	41.8 ± 3.7	39.6 ± 4.1	t = 4.47	<0.001
Prealbumin, mg/L	244 ± 46	223 ± 49	t = 3.47	<0.001

RTOG, Radiation Therapy Oncology Group; Gy, gray.

### 3.4 Univariate analysis of factors associated with severe radiation-induced dermatitis

In the univariate logistic regression analysis, age was positively associated with severe radiation-induced dermatitis (OR = 1.04, 95% CI: 1.01–1.07, P = 0.005), whereas BMI was inversely associated with the outcome (OR = 0.89, 95% CI: 0.81–0.98, P = 0.013). Smoking history (OR = 1.98, 95% CI: 1.15–3.41, P = 0.013), advanced TNM stage (III–IVA vs. I–II; OR = 2.50, 95% CI: 1.44–4.35, P = 0.001), and comorbidities (OR = 1.96, 95% CI: 1.15–3.31, P = 0.013) were also significantly associated with severe dermatitis. Among laboratory and nutritional indicators, albumin (OR = 0.86, 95% CI: 0.80–0.94, P < 0.001) and hemoglobin (OR = 0.97, 95% CI: 0.95–0.99, P = 0.006) were inversely associated with severe dermatitis. C-reactive protein (OR = 1.16, 95% CI: 1.09–1.24, P < 0.001) and neutrophil-to-lymphocyte ratio (OR = 2.03, 95% CI: 1.45–2.85, P < 0.001) were positively associated with the outcome. For treatment-related variables, total radiation dose (OR = 1.10, 95% CI: 1.02–1.19, P = 0.014), radiotherapy duration (OR = 1.09, 95% CI: 1.03–1.16, P = 0.004), concurrent chemotherapy (OR = 2.50, 95% CI: 1.26–4.95, P = 0.008), and irradiation area (OR = 1.19, 95% CI: 1.08–1.31, P < 0.001) were significantly associated with severe dermatitis. Concurrent targeted therapy, cetuximab, nimotuzumab, any immunotherapy, induction immunotherapy, concurrent immunotherapy, and adjuvant/consolidation immunotherapy were not significantly associated with the outcome in the univariate analysis. Sex, alcohol consumption history, pathological type, and dose per fraction were also not significantly associated with severe radiation-induced dermatitis (**Table 3**).

**Table 3:** Univariate logistic regression analysis of factors associated with severe radiation-induced dermatitis.

Variable	OR	95% CI	Wald χ²	P value
Age, per 1-year increase	1.04	1.01–1.07	7.84	0.005
Male sex	1.25	0.73–2.12	0.66	0.415
BMI, per 1 kg/m² increase	0.89	0.81–0.98	6.21	0.013
Smoking history	1.98	1.15–3.41	6.14	0.013
Alcohol consumption history	1.73	0.98–3.06	3.59	0.058
Advanced TNM stage (III–IVA vs. I–II)	2.50	1.44–4.35	10.63	0.001
Undifferentiated non-keratinizing carcinoma vs. other pathological types	1.26	0.70–2.24	0.59	0.441
Comorbidities	1.96	1.15–3.31	6.23	0.013
Albumin, per 1 g/L increase	0.86	0.80–0.94	11.86	<0.001
Hemoglobin, per 1 g/L increase	0.97	0.95–0.99	7.60	0.006
Total radiation dose, per 1 Gy increase	1.10	1.02–1.19	6.05	0.014
Dose per fraction, per 0.1 Gy increase	1.19	0.95–1.48	2.21	0.137
Radiotherapy duration, per 1-day increase	1.09	1.03–1.16	8.44	0.004
Concurrent chemotherapy	2.50	1.26–4.95	6.94	0.008
C-reactive protein, per 1 mg/L increase	1.16	1.09–1.24	21.13	<0.001
Neutrophil-to-lymphocyte ratio, per 1-unit increase	2.03	1.45–2.85	16.54	<0.001
Irradiation area, per 10 cm² increase	1.19	1.08–1.31	11.97	<0.001
Concurrent targeted therapy	1.19	0.49–2.88	0.14	0.707
Cetuximab vs. no targeted therapy	1.19	0.39–3.58	0.09	0.762
Nimotuzumab vs. no targeted therapy	1.19	0.29–4.86	0.06	0.813
Any immunotherapy	1.19	0.55–2.58	0.20	0.653
Induction immunotherapy	1.18	0.43–3.26	0.10	0.747
Concurrent immunotherapy	1.35	0.39–4.74	0.22	0.637
Adjuvant/consolidation immunotherapy	1.18	0.34–4.02	0.07	0.795

OR, odds ratio; CI, confidence interval; BMI, body mass index; TNM, tumor-node-metastasis; Gy, gray.

### 3.5 Multivariable logistic regression analysis of independent factors associated with severe radiation-induced dermatitis

Multivariable logistic regression identified independent factors significantly associated with severe radiation-induced dermatitis. Older age (OR = 1.03, 95% CI: 1.01–1.06, P = 0.017), smoking history (OR = 1.93, 95% CI: 1.11–3.55, P = 0.019), higher total radiation dose (OR = 1.08, 95% CI: 1.01–1.15, P = 0.020), elevated C-reactive protein (OR = 1.12, 95% CI: 1.06–1.20, P < 0.001), higher neutrophil-to-lymphocyte ratio (OR = 1.53, 95% CI: 1.21–1.96, P < 0.001), and larger irradiation area (OR = 1.16, 95% CI: 1.06–1.28, P = 0.002) were independently associated with an increased risk of severe dermatitis. In contrast, higher albumin level was independently associated with a reduced risk of severe radiation-induced dermatitis (OR = 0.89, 95% CI: 0.82–0.95, P = 0.002) (**Table 4**).

**Table 4:** Multivariable logistic regression analysis of factors associated with severe radiation-induced dermatitis.

Variable	β	SE	Wald χ²	OR	95% CI	P value
Age, per 1-year increase	0.031	0.013	5.69	1.03	1.01–1.06	0.017
Smoking history	0.658	0.281	5.48	1.93	1.11–3.55	0.019
Albumin, per 1 g/L increase	-0.118	0.039	9.16	0.89	0.82–0.95	0.002
Total radiation dose, per 1 Gy increase	0.079	0.034	5.40	1.08	1.01–1.15	0.020
C-reactive protein, per 1 mg/L increase	0.112	0.030	13.94	1.12	1.06–1.20	<0.001
Neutrophil-to-lymphocyte ratio, per 1-unit increase	0.426	0.121	12.41	1.53	1.21–1.96	<0.001
Irradiation area, per 10 cm² increase	0.151	0.048	9.89	1.16	1.06–1.28	0.002

OR, odds ratio; CI, confidence interval; SE, standard error.

To assess the potential influence of concurrent chemotherapy, advanced TNM stage, and radiotherapy modality on the multivariable results, additional sensitivity analyses were performed by extending the main multivariable model. The main model included age, smoking history, albumin, total radiation dose, C-reactive protein, neutrophil-to-lymphocyte ratio, and irradiation area. In the extended models, concurrent chemotherapy, advanced TNM stage, and radiotherapy modality were added separately or in combination. The direction and statistical significance of the variables retained in the main model remained generally consistent across all extended models. After additional adjustment, concurrent chemotherapy was not significantly associated with severe radiation-induced dermatitis in Model 2, Model 4, or Model 6. Advanced TNM stage was not significantly associated with severe dermatitis in Model 3, Model 4, or Model 6. Similarly, radiotherapy modality was not significantly associated with severe dermatitis when added alone in Model 5 or when added together with concurrent chemotherapy and advanced TNM stage in Model 6. These results were consistent with the main multivariable model and are presented in **Supplementary Table 2**.

### 3.6 Subgroup analysis and sensitivity analysis

Subgroup analyses were performed according to age group, concurrent chemotherapy status, TNM stage, and nutritional status. Overall, the associations of inflammatory burden, irradiation area, and nutritional indicators with severe radiation-induced dermatitis remained directionally consistent across most strata. Elevated C-reactive protein and larger irradiation area were significantly associated with severe dermatitis in both younger and older patients, with slightly stronger effect sizes in patients aged ≥50 years. Similarly, a higher neutrophil-to-lymphocyte ratio and lower albumin remained significantly associated with severe dermatitis regardless of whether concurrent chemotherapy was administered. Smoking history and total radiation dose showed stronger associations in patients with stage III–IVA disease than in those with stage I–II disease. In addition, the effects of elevated C-reactive protein and larger irradiation area appeared more pronounced in patients with poorer nutritional status (albumin <40 g/L). However, no statistically significant interaction was observed across the predefined subgroups (all interaction P > 0.05), indicating that the identified associations were generally stable (**Supplementary Table 3**).

To evaluate the robustness of the main findings, multiple imputation was performed for missing data. The missing data pattern is shown in **Supplementary Table 4**. After imputation, the direction, magnitude, and statistical significance of the main associations remained largely consistent with the complete-case analysis. Age, smoking history, lower albumin, higher total radiation dose, elevated C-reactive protein, higher neutrophil-to-lymphocyte ratio, and larger irradiation area remained significantly associated with severe radiation-induced dermatitis in the imputed dataset (**Table 5**).

**Table 5:** Sensitivity analysis before and after multiple imputation for missing data.

Variable	Complete-case analysis OR (95% CI)	P value	Multiple imputation analysis OR (95% CI)	P value
Age, per 1-year increase	1.03 (1.01–1.06)	0.017	1.03 (1.01–1.05)	0.014
Smoking history	1.93 (1.11–3.55)	0.019	1.88 (1.09–3.27)	0.023
Albumin, per 1 g/L increase	0.89 (0.82–0.95)	0.002	0.90 (0.84–0.96)	0.003
Total radiation dose, per 1 Gy increase	1.08 (1.01–1.15)	0.020	1.07 (1.01–1.14)	0.024
C-reactive protein, per 1 mg/L increase	1.12 (1.06–1.20)	<0.001	1.11 (1.05–1.18)	<0.001
Neutrophil-to-lymphocyte ratio, per 1-unit increase	1.53 (1.21–1.96)	<0.001	1.49 (1.18–1.88)	0.001
Irradiation area, per 10 cm² increase	1.16 (1.06–1.28)	0.002	1.15 (1.05–1.26)	0.003

OR, odds ratio; CI, confidence interval.

## 4 Discussion

In this retrospective study of 287 patients with nasopharyngeal carcinoma undergoing radiotherapy, severe radiation-induced dermatitis, defined as Radiation Therapy Oncology Group grade ≥3, was independently associated with older age, smoking history, higher total radiation dose, elevated C-reactive protein, higher neutrophil-to-lymphocyte ratio, and larger irradiation area, whereas higher albumin level was independently associated with lower odds of severe dermatitis. These associations were generally consistent across predefined subgroup analyses and remained largely unchanged after multiple imputation, suggesting the internal stability of the main findings. Taken together, these results suggest that clinically significant radiation-induced dermatitis in this setting may be characterized by a multidimensional risk profile involving host-related factors, systemic inflammatory status, nutritional reserve, and treatment-related skin exposure (18, 19). The overall pattern of findings is clinically plausible. Acute radiation dermatitis is among the most frequent toxicities of external-beam radiotherapy, and its burden is particularly relevant in head and neck malignancies because the skin within the treatment field is thin, highly exposed, and often overlaps with areas prone to moisture and friction. Contemporary guidance and recent reviews suggest that radiation-induced epidermal injury involves not only radiation-related epithelial damage but also inflammatory activation, keratinocyte stress responses, immune-cell recruitment, epidermal barrier disruption, and impaired tissue repair (20). This biological framework is consistent with the observed associations with inflammatory markers, nutritional indicators, and irradiation area (21). However, given the retrospective observational design, these findings should be interpreted as associations rather than evidence of causality.

Older age and smoking history remained independently associated with severe radiation-induced dermatitis after adjustment for laboratory and treatment-related variables, suggesting that routinely available host-related characteristics provide information beyond radiotherapy parameters alone. In this cohort, the age effect was modest on a per-year basis, but its persistence in the multivariable model indicates that chronological age may capture a broader vulnerability profile during a multi-week course of fractionated irradiation. Notably, comorbidities were more common in the severe group and were associated with severe dermatitis in the univariate analysis, but they were not retained in the final model. This pattern suggests that the association between age and severe dermatitis was not fully accounted for by the recorded comorbidity status, although age may still integrate unmeasured differences in physiologic reserve, vascular function, and recovery capacity. Smoking history showed a more pronounced association, with an adjusted odds ratio close to 2.0. This finding is clinically relevant because smoking is a readily identifiable exposure before radiotherapy and may reflect a background of impaired tissue oxygenation, microvascular compromise, and delayed epithelial recovery. The association is also consistent with previous head and neck radiotherapy studies reporting smoking-related differences in acute skin toxicity (22). Importantly, smoking should not be interpreted as a deterministic marker of severe dermatitis; rather, it may serve as a practical clinical indicator that helps identify patients who warrant more careful skin assessment during treatment. Together, the age and smoking findings emphasize that severe radiation dermatitis is not explained solely by prescribed dose or treatment field size, but also by patient-level susceptibility that is available for assessment before radiotherapy begins (23).

The treatment-related findings further refine the interpretation of radiation exposure in this cohort. Higher total radiation dose and larger irradiation area remained independently associated with severe dermatitis, whereas dose per fraction did not reach statistical significance. This pattern is coherent with the relatively narrow range of fraction sizes used in standard radiotherapy practice and suggests that cumulative dose and the extent of exposed skin may be more informative than small differences in fraction size. The association with irradiation area is especially relevant in nasopharyngeal carcinoma, where target coverage commonly includes the nasopharynx and bilateral cervical nodal regions. A larger irradiation area may therefore represent broader skin involvement within the treatment field and greater opportunity for clinically meaningful dermatitis to develop. This interpretation is also supported by the relationship between disease burden and treatment planning: patients with more advanced T, N, and TNM stage had higher rates of severe dermatitis in baseline comparisons, and advanced TNM stage was significant in univariate analysis, but it was not independently associated with severe dermatitis after adjustment in the extended models. Similarly, concurrent chemotherapy was more frequent in the severe group and significant in univariate analysis, but it was not significant after adjustment. These findings suggest that the apparent effects of stage and chemotherapy may be partly mediated or confounded by treatment intensity, total dose, irradiation area, and systemic condition. Regarding radiotherapy modality, VMAT was more common in the severe group; however, VMAT versus IMRT was not independently associated with severe dermatitis in the extended sensitivity models (24). Therefore, the observed unadjusted difference in modality may reflect case complexity, target geometry, or field characteristics rather than an independent modality effect. In future radiotherapy-technique comparisons, proton therapy may also be relevant because of its distinct normal-tissue dose distribution, particularly when detailed skin-dose and dose-volume parameters are available (25).

Systemic inflammatory and nutritional indicators provided additional information beyond demographic and radiotherapy-related variables. In the multivariable model, both C-reactive protein and neutrophil-to-lymphocyte ratio remained independently associated with severe radiation-induced dermatitis, and these associations were generally stable in subgroup analyses and after multiple imputation. In addition, patients in the severe dermatitis group showed a broader inflammatory profile, including higher white blood cell and neutrophil counts, higher erythrocyte sedimentation rate, and lower lymphocyte counts. These findings suggest that severe dermatitis may be more likely to occur in patients with a higher systemic inflammatory burden at baseline or during treatment (26, 27). However, CRP and NLR should be interpreted as clinically accessible inflammatory indicators rather than definitive mechanistic biomarkers, because circulating inflammatory markers may reflect tumor burden, concurrent treatment, comorbid conditions, infection, nutritional status, or other host-related factors. Albumin was inversely associated with severe dermatitis in both the primary multivariable model and the imputed analysis. This association should not be interpreted as evidence of a direct protective effect; rather, albumin may represent a composite marker of nutritional reserve, systemic catabolism, and inflammatory status. The subgroup findings further suggested that the associations of inflammatory burden with severe dermatitis were numerically more pronounced among patients with lower albumin levels, although no statistically significant interaction was observed (28, 29). This pattern is clinically relevant because inflammation and impaired nutritional status often coexist during radiotherapy and may jointly characterize patients with lower tolerance to treatment-related skin toxicity (30). Therefore, baseline CRP, NLR, and albumin may serve as practical and routinely available parameters that complement clinical and radiotherapy-related variables when evaluating the likelihood of severe radiation-induced dermatitis in patients with nasopharyngeal carcinoma.

This study has several limitations. First, the retrospective single-center design may have introduced selection bias and residual confounding; therefore, the observed associations should not be interpreted as evidence of causality. Second, although radiation-induced dermatitis was assessed using standardized RTOG criteria, grading still partly depends on clinical judgment and the quality of medical documentation. Third, several potentially relevant factors were not included in the final analysis, including detailed skin dose-volume parameters, topical skin-care adherence, environmental exposure, skin phenotype, and patient-reported symptoms. The absence of detailed skin dosimetric data also limited further evaluation of how irradiation area, dose distribution, and radiotherapy technique jointly relate to severe dermatitis. Fourth, proton therapy was not used in this cohort, so its potential association with radiation-induced dermatitis could not be directly assessed. Finally, although multiple imputation supported the robustness of the main findings, external validation was not performed. Future studies should validate these findings in larger, prospective, multicenter cohorts with standardized and longitudinal dermatitis assessment. Such designs would allow more precise characterization of dermatitis onset, progression, and recovery, while reducing heterogeneity in toxicity evaluation. Future studies should validate these findings in larger, prospective, multicenter cohorts with standardized longitudinal dermatitis assessment. Detailed skin dose-volume parameters and radiotherapy planning data should be incorporated to clarify the relative contributions of total dose, irradiation area, and radiotherapy modality. Comparative studies involving IMRT, VMAT, and proton therapy may further characterize technique-specific skin toxicity profiles. In addition, serial inflammatory and nutritional markers may help determine whether dynamic changes improve risk stratification beyond baseline measurements. Ultimately, externally validated models integrating clinical, laboratory, and radiotherapy-related parameters may support individualized monitoring of clinically significant radiation-induced dermatitis.

## 5 Conclusion

In conclusion, severe radiation-induced dermatitis in patients with nasopharyngeal carcinoma undergoing radiotherapy was independently associated with older age, smoking history, higher total radiation dose, elevated inflammatory markers, and larger irradiation area, whereas higher albumin level was inversely associated with severe dermatitis. These findings suggest that integrating clinical, laboratory, and treatment-related factors may help inform early risk stratification and targeted preventive management during radiotherapy.

## Data Availability

The raw data supporting the conclusions of this article will be made available by the authors, without undue reservation.
