# Supplementary material for: Risk factors for severe radiation-induced dermatitis in nasopharyngeal carcinoma patients undergoing radiotherapy
Source: Front Oncol. 2026 Jul 27;16:1851374. doi: 10.3389/fonc.2026.1851374 (PMC13453732; doi:10.3389/fonc.2026.1851374)
Supplement: Supplementary file 1 [file Table1.docx]

Supplementary Table S1. Incidence, severity distribution, and onset time of radiation-induced dermatitis

| Variable | Overall cohort (n = 287) |
| --- | --- |
| Severity distribution of radiation-induced dermatitis, n (%) |  |
| RTOG grade 0 | 28 (9.8) |
| RTOG grade 1 | 76 (26.5) |
| RTOG grade 2 | 97 (33.8) |
| RTOG grade 3 | 82 (28.6) |
| RTOG grade 4 | 4 (1.4) |
| Incidence of radiation-induced dermatitis, n (%) |  |
| Any radiation-induced dermatitis, RTOG grade ≥1 | 259 (90.2) |
| Severe radiation-induced dermatitis, RTOG grade ≥3 | 86 (30.0) |
| Onset time of radiation-induced dermatitis |  |
| Time from initiation of radiotherapy to first recorded RTOG grade ≥1 dermatitis among patients with RTOG grade ≥1 dermatitis, days | 24.8 ± 6.7 |
| Median time to first recorded RTOG grade ≥1 dermatitis among patients with RTOG grade ≥1 dermatitis, days | 25.0 (20.0–29.0) |
| Time from initiation of radiotherapy to first recorded RTOG grade ≥3 dermatitis among patients with RTOG grade ≥3 dermatitis, days | 37.6 ± 6.3 |
| Median time to first recorded RTOG grade ≥3 dermatitis among patients with RTOG grade ≥3 dermatitis, days | 38.0 (33.0–42.0) |

Abbreviations: RTOG, Radiation Therapy Oncology Group.

Note: Values are presented as n (%), mean ± SD, or median (interquartile range), as appropriate. Percentages for RTOG grade distribution and incidence were calculated using the total cohort as the denominator (n = 287). The onset time of RTOG grade ≥1 dermatitis was calculated among patients who developed any dermatitis (n = 259), whereas the onset time of RTOG grade ≥3 dermatitis was calculated among patients who developed severe dermatitis (n = 86).

Supplementary Table S2. Sensitivity analysis of multivariable models additionally including concurrent chemotherapy, advanced TNM stage, and radiotherapy modality

| Variable | Model 1: Main model OR (95% CI), P value | Model 2: Main model + concurrent chemotherapy OR (95% CI), P value | Model 3: Main model + advanced TNM stage OR (95% CI), P value | Model 4: Main model + concurrent chemotherapy + advanced TNM stage OR (95% CI), P value | Model 5: Main model + radiotherapy modality OR (95% CI), P value | Model 6: Main model + concurrent chemotherapy + advanced TNM stage + radiotherapy modality OR (95% CI), P value |
| --- | --- | --- | --- | --- | --- | --- |
| Age, per 1-year increase | 1.03 (1.01–1.06), 0.017 | 1.03 (1.01–1.06), 0.019 | 1.03 (1.01–1.06), 0.021 | 1.03 (1.01–1.06), 0.024 | 1.03 (1.01–1.06), 0.020 | 1.03 (1.00–1.06), 0.029 |
| Smoking history | 1.93 (1.11–3.55), 0.019 | 1.90 (1.08–3.49), 0.025 | 1.86 (1.05–3.43), 0.031 | 1.82 (1.02–3.38), 0.041 | 1.88 (1.06–3.45), 0.028 | 1.79 (1.00–3.34), 0.049 |
| Albumin, per 1 g/L increase | 0.89 (0.82–0.95), 0.002 | 0.89 (0.83–0.96), 0.003 | 0.90 (0.83–0.96), 0.004 | 0.90 (0.83–0.97), 0.005 | 0.89 (0.83–0.96), 0.003 | 0.90 (0.83–0.97), 0.006 |
| Total radiation dose, per 1 Gy increase | 1.08 (1.01–1.15), 0.020 | 1.08 (1.01–1.15), 0.024 | 1.07 (1.01–1.15), 0.029 | 1.07 (1.00–1.14), 0.046 | 1.07 (1.01–1.15), 0.032 | 1.06 (1.00–1.14), 0.048 |
| C-reactive protein, per 1 mg/L increase | 1.12 (1.06–1.20), <0.001 | 1.12 (1.05–1.19), <0.001 | 1.11 (1.05–1.19), <0.001 | 1.11 (1.05–1.18), <0.001 | 1.11 (1.05–1.19), <0.001 | 1.10 (1.04–1.18), <0.001 |
| Neutrophil-to-lymphocyte ratio, per 1-unit increase | 1.53 (1.21–1.96), <0.001 | 1.51 (1.19–1.94), 0.001 | 1.49 (1.17–1.91), 0.001 | 1.47 (1.15–1.89), 0.002 | 1.50 (1.18–1.92), 0.001 | 1.45 (1.13–1.87), 0.003 |
| Irradiation area, per 10 cm² increase | 1.16 (1.06–1.28), 0.002 | 1.15 (1.05–1.27), 0.003 | 1.14 (1.04–1.26), 0.005 | 1.13 (1.03–1.25), 0.011 | 1.14 (1.04–1.26), 0.005 | 1.12 (1.01–1.24), 0.027 |
| Concurrent chemotherapy | — | 1.34 (0.65–2.78), 0.428 | — | 1.24 (0.58–2.65), 0.578 | — | 1.18 (0.54–2.58), 0.667 |
| Advanced TNM stage, III–IVA vs. I–II | — | — | 1.39 (0.70–2.77), 0.351 | 1.31 (0.63–2.73), 0.469 | — | 1.25 (0.58–2.69), 0.572 |
| Radiotherapy modality, VMAT vs. IMRT | — | — | — | — | 1.36 (0.71–2.62), 0.358 | 1.27 (0.65–2.55), 0.491 |

Abbreviations: OR, odds ratio; CI, confidence interval; TNM, tumor-node-metastasis; VMAT, volumetric modulated arc therapy; IMRT, intensity-modulated radiotherapy.

Model 1: age, smoking history, albumin, total radiation dose, C-reactive protein, neutrophil-to-lymphocyte ratio, and irradiation area.

Model 2: Model 1 plus concurrent chemotherapy.

Model 3: Model 1 plus advanced TNM stage.

Model 4: Model 1 plus concurrent chemotherapy and advanced TNM stage.

Model 5: Model 1 plus radiotherapy modality.

Model 6: Model 1 plus concurrent chemotherapy, advanced TNM stage, and radiotherapy modality.

Supplementary Table S3. Subgroup analyses of factors associated with severe radiation-induced dermatitis

| Subgroup | Comparison / Variable | OR | 95% CI | Interaction P value | P value within subgroup |
| --- | --- | --- | --- | --- | --- |
| Age group |  |  |  | 0.118 |  |
| <50 years | Elevated C-reactive protein, per 1 mg/L increase | 1.10 | 1.03–1.18 |  | 0.006 |
| ≥50 years | Elevated C-reactive protein, per 1 mg/L increase | 1.15 | 1.08–1.24 |  | <0.001 |
| <50 years | Higher irradiation area, per 10 cm² increase | 1.12 | 1.01–1.24 |  | 0.031 |
| ≥50 years | Higher irradiation area, per 10 cm² increase | 1.20 | 1.08–1.34 |  | 0.001 |
| Concurrent chemotherapy |  |  |  | 0.214 |  |
| No | Higher neutrophil-to-lymphocyte ratio, per 1-unit increase | 1.41 | 1.05–1.90 |  | 0.022 |
| Yes | Higher neutrophil-to-lymphocyte ratio, per 1-unit increase | 1.58 | 1.21–2.07 |  | 0.001 |
| No | Lower albumin, per 1 g/L decrease | 1.09 | 1.01–1.18 |  | 0.028 |
| Yes | Lower albumin, per 1 g/L decrease | 1.14 | 1.06–1.23 |  | <0.001 |
| TNM stage |  |  |  | 0.167 |  |
| Stage I–II | Smoking history | 1.62 | 0.79–3.31 |  | 0.188 |
| Stage III–IVA | Smoking history | 2.14 | 1.19–3.84 |  | 0.011 |
| Stage I–II | Higher total radiation dose, per 1 Gy increase | 1.05 | 0.96–1.15 |  | 0.284 |
| Stage III–IVA | Higher total radiation dose, per 1 Gy increase | 1.11 | 1.03–1.20 |  | 0.008 |
| Nutritional status |  |  |  | 0.093 |  |
| Albumin ≥40 g/L | Higher C-reactive protein, per 1 mg/L increase | 1.09 | 1.02–1.17 |  | 0.010 |
| Albumin <40 g/L | Higher C-reactive protein, per 1 mg/L increase | 1.16 | 1.08–1.25 |  | <0.001 |
| Albumin ≥40 g/L | Higher irradiation area, per 10 cm² increase | 1.11 | 1.01–1.22 |  | 0.029 |
| Albumin <40 g/L | Higher irradiation area, per 10 cm² increase | 1.21 | 1.09–1.35 |  | <0.001 |

Abbreviations: OR, odds ratio; CI, confidence interval; TNM, tumor-node-metastasis.

Supplementary Table S4. Missing data pattern of variables included in the analysis

| Variable | Missing values, n (%) | Handling method |
| --- | --- | --- |
| Severe radiation-induced dermatitis | 0 (0.0) | Complete |
| Age | 0 (0.0) | Complete |
| Sex | 0 (0.0) | Complete |
| BMI | 3 (1.0) | Multiple imputation |
| Smoking history | 0 (0.0) | Complete |
| Alcohol consumption history | 2 (0.7) | Multiple imputation |
| TNM stage | 0 (0.0) | Complete |
| Concurrent chemotherapy | 0 (0.0) | Complete |
| Concurrent targeted therapy | 0 (0.0) | Complete |
| Immunotherapy | 0 (0.0) | Complete |
| Total radiation dose | 0 (0.0) | Complete |
| Irradiation area | 4 (1.4) | Multiple imputation |
| C-reactive protein | 6 (2.1) | Multiple imputation |
| Neutrophil-to-lymphocyte ratio | 5 (1.7) | Multiple imputation |
| Albumin | 4 (1.4) | Multiple imputation |
| Hemoglobin | 3 (1.0) | Multiple imputation |
| Prealbumin | 12 (4.2) | Multiple imputation |
